# Energy Balance, Myostatin, and GILZ: Factors Regulating Adipocyte Differentiation in Belly and Bone

**DOI:** 10.1155/2007/92501

**Published:** 2007-11-25

**Authors:** Xingming Shi, Mark Hamrick, Carlos M. Isales

**Affiliations:** ^1^Institute of Molecular Medicine and Genetics, Medical College of Georgia, GA 30912, USA; ^2^Department of Pathology, Medical College of Georgia Hospital, GA 30912, USA; ^3^Department of Cellular Biology and Anatomy, Medical College of Georgia, GA 30912, USA; ^4^Department of Orthopaedic Surgery, Medical College of Georgia, GA 30912, USA

## Abstract

Peroxisome proliferator-activated receptor gamma (PPAR-γ) belongs to the nuclear hormone receptor subfamily of transcription factors. PPARs are expressed in key target tissues such as liver, fat, and muscle and thus they play a major role in the regulation of energy balance. Because of PPAR-γ's role in energy balance, signals originating from the gut (e.g., GIP), fat (e.g., leptin), muscle (e.g., myostatin), or bone (e.g., GILZ) can in turn modulate PPAR expression and/or function. Of the two PPAR-γ isoforms, PPAR-γ2 is the key regulator of adipogenesis and also plays a role in bone development. Activation of this receptor favors adipocyte differentiation of mesenchymal stem cells, while inhibition of PPAR-γ2 expression shifts the commitment towards the osteoblastogenic pathway. Clinically, activation of this receptor by antidiabetic agents of the thiazolidinedione class results in lower bone mass and increased fracture rates. We propose that inhibition of PPAR-γ2 expression in mesenchymal stem cells by use of some of the hormones/factors mentioned above may be a useful therapeutic strategy to favor bone formation.

## 1. INTRODUCTION

The peroxisome proliferator-activated receptor gamma (PPAR-*γ*) family of transcription factors belongs to the nuclear hormone receptor subfamily of transcription factors that can bind to specific DNA response elements in the regulatory regions of target genes. Other isotypes of this family include PPAR alpha (PPAR-*α*) and PPAR beta/delta (PPAR-*β*/-*δ*). Each of these isotypes is encoded by a different gene and has different functions and different tissue distribution [1]. Many excellent reviews have been published recently on the regulation of nuclear receptors belonging to the PPAR family [2–12] and the reader is referred to one of these reviews for an overview on these nuclear receptors.

**Figure 1:**
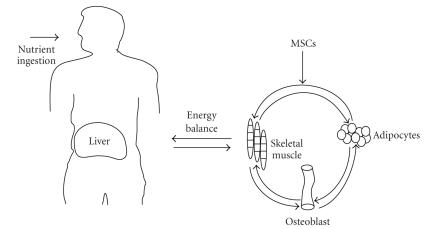
*Nutrition and tissue-generated hormonal signals modulate mesenchymal stem cell differentiation*. Hormonal signals generated upon nutrient ingestion impact the organism's energy balance, thus favoring anabolic versus catabolic activities. In turn, hormonal signals generated by target tissues such as muscle, bone, and fat modulate MSC differentiation into adipocytes or osteoblasts.

The current review more narrowly focuses on PPAR-*γ*2, its role in bone formation, and its regulation by the energy state. Bone, like other tissues in the body, requires a positive energy balance to grow. However, even with a positive energy balance, bone progenitor cells can differentiate into osteoblast, adipocyte, or muscle cells. PPAR-*γ*2 is a key regulator of this differentiation step in bone marrow progenitor cells. Tissues in the body important in regulating this energy balance include skeletal muscle, adipocytes, and liver (see Figure 1). The crosstalk between these target tissues occurs via both central nervous system (CNS) output and peripheral hormones from enteric, pancreatic, or adipocytic sources. Studies from our laboratories have focused on enteric hormones like glucose-dependent insulinotropic peptide (GIP), adipocytic hormones like leptin, and skeletal muscle-derived factors such as myostatin in addition to transcriptional regulators such as the glucocorticoid-induced leucine zipper (GILZ) in the regulation of osteoblast/adipocyte differentiation from bone marrow progenitor cells. The impact of changes in the organism's energy balance on mesenchymal stem cell differentiation will be discussed in more detail below.

## 2. PEROXISOME PROLIFERATOR-ACTIVATED RECEPTOR

PPAR-*γ* is highly expressed in adipose tissue although it is also expressed in other tissues including skeletal muscle, intestine, endothelium, prostate, and white blood cells [13]. The gene for PPAR-*γ* is localized on chromosome 3 [13] and there are two protein products, PPAR-*γ*1 and PPAR-*γ*2, which are isoforms transcribed from the same gene with different promoter usage [14, 15]. PPAR-*γ*2 is predominantly expressed in adipose tissue, while PPAR-*γ*1 is more widely expressed [10]. Of these two isoforms, PPAR-*γ*2 is a key regulator of adipogenesis [16–18] and is expressed at an early stage of the adipogenesis program [19]. PPAR-*γ*2 can activate a battery of genes necessary for lipid metabolism, including lipoprotein lipase (LPL) [20], phosphoenolpyruvate carboxykinase (PEPCK) [21], fatty acid-binding and transport proteins [22], and stearoyl-CoA desaturase-1 (SCD-1) [23]. Functional PPAR response elements (PPREs) have been identified in the promoter regions of these genes. In addition, activation of PPAR-*γ*2 by its ligand [24, 25] induces cell cycle withdrawal and terminal adipocyte differentiation in a variety of mesenchymal cell lines [26–29]. Thus, the pivotal regulatory role that PPAR-*γ*2 plays in adipocyte differentiation is recognized by its early and tissue-specific expression [19] and its ability to direct fibroblasts and myoblasts to differentiate into adipocytes when it is ectopically expressed in these cells [28, 30].

Most importantly, a recent in vivo study has demonstrated that PPAR-*γ* insufficiency in mice (PPAR-*γ* ^+/−^) results in a dramatic decrease (by 50%) in adipogenesis with a concomitant increase in osteogenesis through osteoblast formation from marrow progenitors [31]. This study suggests the possibility of interrupting the PPAR-*γ* pathway as a novel treatment of osteoporosis.

## 3. MESENCHYMAL STEM CELLS

Mesenchymal stem cells (MSCs) are multipotent cells that, under appropriate culture conditions, can differentiate into multiple cell lineages, including osteoblasts, myoblasts, and adipocytes [32–35]. Considerable evidence has shown that the commitment between osteoblast and adipocyte lineages from MSCs is reciprocal, that is, when the adipogenic pathway is blocked, MSCs enter the osteogenic pathway, and vice versa [36–40]. Increased marrow adipogenesis negatively impacts bone formation because mesenchymal precursor cells are directed towards the adipocyte lineage rather than to the osteoblast lineage [41, 42]. Marrow adipocytes can also inhibit osteoblast proliferation in vitro, and adipocytes secrete factors such as IL-6 and TNF*α* [43] that stimulate the differentiation of the bone-resorbing cells, osteoclasts [44]. The negative impact of marrow adipogenesis on bone health is further indicated by the fact that bone formation rate is inversely correlated with adipocyte number in bone biopsies of adult men and women [45], and women with osteoporosis have higher number of marrow adipocytes than those with healthy bone [46].

Clinically, much recent attention has focused on drugs belonging to the thiazolidinedione class. These medications (e.g., rosiglitazone and pioglitazone) are PPAR agonists used to treat patients with diabetes mellitus, and have recently been associated with impaired bone quality, increased marrow fat, and increased fracture rates [47, 48].

Understanding the regulators of MSC differentiation between fat and bone has gained increasing importance with increasing human longevity since, as humans age, the number of adipocytes increases and the number of osteoblasts decreases resulting in weakened bone, age-related osteoporosis, and fragility fractures. Because of the importance of PPAR-*γ*2 in MSC differentiation into adipocytes or osteoblasts, we will briefly discuss some of the regulators of the PPAR-*γ*2 receptor.

## 4. REGULATION OF THE PPAR-*γ* RECEPTOR

**Figure 2:**
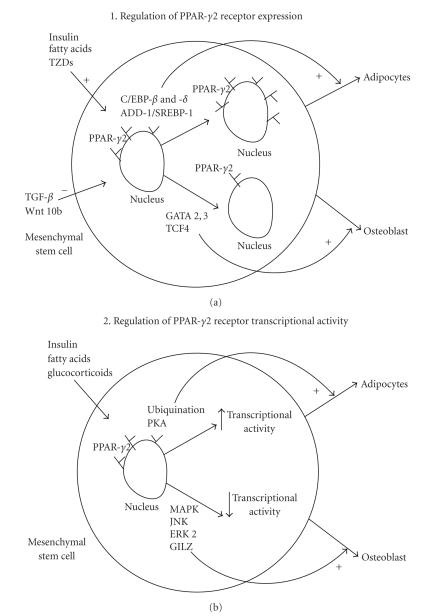
*PPAR-*γ*2 action is modulated by either changes in receptor expression or transcriptional activity*. (a) Natural (insulin, long-chain fatty acids, or eicosanoids) or synthetic ligands (TZDs) to the PPAR-*γ*2 receptor can either increase or decrease receptor expression resulting in increased adipocytic or increased osteoblastic differentiation, respectively. (b) It is also possible to stimulate or inhibit PPAR-*γ*2 transcriptional activity resulting in either increased adipocytic or osteoblastic differentiation, respectively.

Regulation of the PPAR-*γ* receptor activity can occur via (1) changes in receptor expression levels or (2) changes in transcriptional activity (see Figure 2).

A number of transcription factors can either positively or negatively modulate PPAR-*γ* receptor expression in adipocytes [49]. Major transcription factors activating PPAR-*γ* receptor expression include CCAAT/enhancer-binding protein (C/EBP) family of transcription factors and these have been reviewed extensively elsewhere [2, 50]. Although GATA-1, -2, Wnt (Wnt 10b), epidermal growth factor (EGF), platelet-derived growth factor (PDGF), and transforming growth factor beta (TGF-*β*) play important roles in PPAR-*γ* regulation, they will not be discussed in this review.

PPAR-*γ* receptor transcriptional activity is regulated by two distinct processes: repression of receptor activity by phosphorylation (by kinases such as mitogen-activated protein, MAP kinases, which activate Jun N-terminal kinase, or JNK, and extracellular signal-regulated kinase 2, or ERK-2) and increased receptor activity by ubiquitination. Agonists for the nuclear PPAR-*γ* receptor include protein kinase A, natural fatty acids, eicosanoids, and oxidized lipoproteins. Less well studied are negative regulators of the nuclear PPAR-*γ* receptor. Activators of MAPK and thus inhibitors of PPAR-*γ* receptor transcriptional activity include growth factors like epidermal growth factor (EGF), platelet-derived growth factor (PDGF), transforming growth factor *β*'s (TGF-*β*'s) 1 and 2, and GILZ. Both insulin and glucocorticoid induce the expression of C/EBP-*β* and -*δ*, which in turn induce the expression of PPAR-*γ* and C/EBP-*α* and initiate the adipogenesis program.

## 5. NUTRITION-RELATED HORMONES

Enteric hormones represent the mechanism by which ingested nutrients are distributed to the various tissues in the body so as to maximize their utilization. These hormones play a key role in regulating the energy balance, in part through modulation of PPAR expression. In fact, elevation of incretin hormones, through use of inhibitors of the enzyme that breaks them down (DPP-IV inhibitors), has been shown to increase PPAR expression in the kidney [51].

Nutritional hormones are also known to be important in bone turnover as evidenced by the fact that as soon as a meal is ingested, bone breakdown is suppressed. Many nutrition-related hormones have been shown to have effects on bone turnover through in vitro or in vivo studies including (a) *Intestinal Hormones* such as (1) GIP, (2) Ghrelin, and (3) Glucagon-like peptide (GLP-2); (b) *Pancreatic Hormones* such as (1) Insulin, (2) Amylin, (3) Adrenomedullin, and (4) Preptin; (c) *Adipocyte-secreted Hormones* such as (1) Leptin, (2) Adiponectin, and (3) Resistin, as recently reviewed by Clowes et al. [52] and Reid et al. [53]. For purposes of this review, we will focus more extensively on GIP and leptin but discuss these other hormones briefly below.

### 5.1. Intestinal hormones

Ghrelin is a 28-amino acid peptide expressed predominantly in the gastric epithelium and small intestine, though it is also expressed to a lower extent in the brain, pancreatic islets, adrenal cortex, kidney, and bone [54]. Ghrelin’s physiologic function is to stimulate growth hormone secretion, and systemic elevations of ghrelin stimulate food intake and weight gain. Ghrelin’s systemic effects on energy metabolism appear to oppose those of leptin. Ghrelin receptors are expressed on osteoblasts and ghrelin stimulates osteoblastic proliferation and differentiation [55, 56]. In addition, intraperitoneal infusion of ghrelin for four weeks resulted in significant increases in bone mineral density in Sprague-Dawley rats [55]. In humans, the data supporting a role for ghrelin in bone turnover are less clear. Ghrelin levels have a significant negative correlation with markers of bone breakdown at baseline, although not with bone mineral density, and ghrelin infusion has no acute effect on these markers [57, 58].

GLP-2 is a 33-amino acid peptide expressed mainly in the L cells of the small intestine. GLP-2 is secreted in response to nutrient ingestion and its physiologic function appears to be to regulate intestinal motility and stimulate intestinal cell growth; it is also antiapoptotic [59]. GLP-2 receptors are expressed in osteoclasts and the administration of GLP-2 to human subjects inhibits bone resorption and increases bone mass [60–62].

### 5.2. Pancreatic hormones

Insulin has long been considered the main anabolic hormone, stimulating bone formation in vitro. However, in vivo, although insulin infusion is known to decrease markers of bone breakdown, this effect is only about 30% of the decline in resorption markers that occurs postprandially. In fact, it has been suggested that this effect is due to hypoglycemia and the attendant impairment in skeletal cellular activity rather than to a direct antiresorptive effect [63].

Amylin is a 37-amino acid hormone cosecreted from the pancreatic *β* cells with insulin in response to a meal. Amylin lowers serum calcium, inhibits bone resorption, and increases bone mass in mice [64–66].

Adrenomedullin is a 52-amino acid peptide related to amylin; it is expressed in the adrenal medulla, vasculature brain, kidney, and bone [67]. Adrenomedullin stimulates osteoblastic proliferation and injection of adrenomedullin to mice increases bone formation and strength without a major effect on bone breakdown [68, 69].

Preptin is a 37-amino acid peptide cosecreted from the pancreatic islet with amylin and insulin. Preptin stimulated osteoblastic proliferation, and the daily injection of this peptide for five days over the calvaria resulted in increased bone area and mineralized surface through increased bone formation rather than through inhibition of bone breakdown [70].

### 5.3. Adipocytic hormones

Adiponectin is a 247-amino acid protein strongly expressed in mature adipocytes (particularly in subcutaneous versus visceral adipocytes) and the levels correlate with the degree of differentiation [71]. Thus, PPAR-*γ* agonists (e.g., thiazolidinediones) are potent stimulators of adiponectin expression. Adiponectin suppresses both cell proliferation and release of other inflammatory cytokines [71]. Both the adiponectin protein and its receptor are expressed in osteoblasts and osteoclasts, and its effects on bone turnover are complex [72, 73]. In humans, adiponectin levels have been shown to be negatively correlated with bone mineral density [74], particularly in postmenopausal female patients [75].

Resistin is a 137-amino acid protein secreted from adipocytes [76]. In addition to adipocytes, resistin is also expressed in pancreas, brain, and bone marrow. In the adipocyte, resistin expression is regulated by PPAR-*γ* with PPAR-*γ* agonists such as rosiglitazone resulting in an inhibition of resistin expression [77]. Resistin secretion results in insulin resistance. In the bone, resistin is expressed in osteoblast, osteoclast, and mesenchymal stem cells [76] and resistin levels are negatively correlated with bone mineral density [78].

### 5.4. Glucose-dependent insulinotropic peptide

There are two major intestinal hormones that potentiate glucose-induced insulin secretion (incretin effect), that is, GIP and glucagon-like peptide-1 (GLP-1). GLP-1 receptors are not present in bone cells [79]. GIP was first identified in the 1970’s as a hormone secreted by cells in the enteric endocrine system (K cells) in the proximal small intestine. Because this 42-amino acid peptide was found to inhibit gastric acid secretion, it was initially named gastric inhibitory peptide (GIP) [80]. Subsequent studies demonstrated that GIP effects on inhibiting gastric acid secretion did not occur at physiological concentrations, in contrast to GIPs effect on potentiating glucose-induced insulin secretion. Thus GIP's name was changed to glucose-dependent insulinotropic peptide. Our data and resulting publications demonstrate that GIP also serves as an important anabolic signal for bone, stimulating bone formation and inhibiting bone breakdown. To summarize our GIP data in vitro, (1) GIP receptors are present in both osteoblasts and osteoclasts [81, 82]; (2) in osteoblasts, GIP increases collagen type I synthesis and increases alkaline phosphatase activity [81]; (3) in osteoblasts, GIP stimulates proliferation [81]; and (4) in osteoclasts, GIP inhibits PTH-induced long bone resorption and decreases osteoclastic resorption pit depth [82]. In in vivo studies, (5) GIP receptor knockout mice have a lower bone mass, decreased serum markers of bone formation, and increased markers of bone breakdown [83], consistent with data published by others, [84] and (6) GIP-overexpressing transgenic mice have increased bone mass, lower serum markers of bone breakdown, and increased markers of bone formation [85].

The GIP receptor knockout mouse was developed by Miyawaki and colleagues [86], and it is an interesting animal model linking nutritional hormones and bone formation. These knockout mice are not different from control mice in their weight, basal insulin, glucose levels, or in their insulin response to an intraperitoneal glucose tolerance test. However, blood glucose levels, in response to an oral glucose tolerance test or a high-fat diet, are higher in the knockout mice compared to controls, and the higher blood glucose levels in the knockout mice are associated with lower insulin levels. A subsequent report, also by Miyawaki et al. [87], demonstrates that although normal mice fed a high-fat diet gained weight, GIP receptor knockout mice were protected from the large weight gain associated with this diet. Interestingly, GIPR knockout mice fed a high-fat diet had lower leptin levels than control mice fed a high-fat diet (2-fold increase from basal in GIPR^−^/^−^ versus 4-fold increase in WT). Furthermore, if ${\text{GIPR}}^{\text{-}}{/}^{\text{-}}$ mice are crossed with the obese mouse model ${\text{Lep}}^{ob}$/${\text{Lep}}^{ob}$ to generate double homozygous mice, these double homozygous mice are partially protected from the weight gain seen in the ${\text{Lep}}^{ob}$/${\text{Lep}}^{ob}$ mice (GIPR^−^/^−^/${\text{Lep}}^{ob}$/${\text{Lep}}^{ob}$ had a 23% lower body weight). The authors conclude that under normal conditions an excessive amount of fat in the diet leads to GIP hypersecretion; this in turn leads to more adiposity, resulting in obesity and insulin resistance.

In our studies, we found that if the GIP receptor is downregulated, bone mass decreases and bone marrow adipocyte content increases [85]. These findings would suggest the possibility of direct GIP effects on MSCs. In fact, we have demonstrated that GIP receptors are present on MSCs and that stimulation of these cells with GIP promotes osteoblastic differentiation [88].

## 6. LEPTIN

The cytokine-like hormone leptin is recognized as a powerful regulator of appetite and energy balance [89]. Adipocytes are the primary source of leptin in the body and as such leptin plays an important role as a signal of energy status to the brain [90]. Leptin produced by peripheral body fat enters the circulation and crosses the blood-brain barrier to reach leptin receptors located in the hypothalamus. Leptin binding to the long form of the leptin receptor induces the expression of anorexigenic neuropeptides such as cocaine-amphetamine-related transcript (CART) and alpha-melanocyte-stimulating hormone (*α*-MSH), and suppresses the activity of orexigenic genes such as neuropeptide Y (NPY) and agouti-related peptide (AgRP), that are involved in regulating food intake [91]. Leptin also regulates sympathetic outflows and functions as a beta-adrenergic agonist [92], and intrahypothalamic injections of leptin induce apoptosis of adipocytes in both peripheral fat and bone marrow [93]. Adipocytes express beta-adrenergic receptors, particularly beta 3 [94], and activation of these receptors can induce apoptosis through activation of a tyrosine kinase pathway [95].

Leptin also appears to regulate adipocyte populations in bone marrow directly, in addition to the central effects of leptin on adipocyte apoptosis. Leptin-deficient ob/ob mice show a significant increase in bone marrow adipocytes compared to lean mice [96], and peripheral leptin injections decrease the population of bone marrow adipocytes in ob/ob mice and increase bone formation [97]. As discussed above, the loss of bone marrow adipocytes with peripheral leptin treatment may be a centrally mediated effect, but the increased osteogenic differentiation and increased endocortical bone formation are more consistent with a direct effect of leptin on osteogenic differentiation [89]. Bone marrow stromal cells (BMSCs) express leptin receptors, and leptin binding increases the expression of osteogenic genes and directs BMSCs to the osteogenic rather than the adipogenic pathway [98]. In studies by Thomas et al. [98], leptin did not alter PPAR-*γ* or Cbfa-1 expression despite increasing osteogenesis and decreasing adipogenesis presumably by acting at a later stage in osteoblast differentiation. Bone marrow adipocytes secrete leptin themselves [99], raising the possibility that leptin may play a role in autocrine or paracrine signaling within the bone marrow microenvironment. We have found that age-associated bone loss in mice is associated with decreased serum leptin [100], and as noted earlier, aging is associated with bone loss and an increased accumulation of bone marrow adipocytes. These data suggest that leptin treatment, in conditions of increased leptin sensitivity (see below), may have significant potential for increasing bone formation and decreasing marrow adipogenesis with aging.

## 7. MYOSTATIN

Myostatin was initially identified as a factor regulating myogenic differentiation because its expression was localized to developing skeletal muscle, and because myostatin loss-of-function was observed to have dramatic effects on muscle mass in mice. It was, however, also noted that mice lacking myostatin showed decreased body fat [101, 102], and myostatin deficiency decreased adiposity in leptin-deficient ob/ob mice [102]. This was thought to be an indirect effect of the increased muscle mass on metabolism. Since that time, we and others have found that myostatin deficiency inhibits adipogenesis in vivo, even when mice are fed a high-fat diet [103]. Transgenic overexpression of myostatin propeptide, which inhibits myostatin signaling, also inhibits body fat gain with a high-fat diet [104]. Similar alterations in myostatin signaling are associated with changes in body fat among humans. A child with a naturally occurring mutation in the myostatin gene was shown to have increased muscle mass as well as decreased subcutaneous fat [105]. Weight loss in morbidly obese subjects was associated with significant downregulation of myostatin mRNA in muscle biopsies, suggesting a role for myostatin in energy partitioning between protein and fat [106].

Although the in vivo data consistently show that myostatin has an adipogenic effect, and that myostatin deficiency has an anti-adipogenic effect, the in vitro data are less clear. Myostatin has been observed to promote adipogenesis in multipotential mouse C3H 10T (1/2) mesenchymal stem cells [107], but myostatin can also inhibit adipocyte differentiation in 3T3-L1 mouse preadipocytes [108] and inhibit BMP-7-mediated adipogenesis by binding to the same receptor as BMP-7, the activin IIB (ActRIIB) receptor [109]. These data suggest that the decreased fat mass of myostatin-deficient animals is simply an indirect effect of increased muscle mass since other mouse models showing increased muscle mass, such as transgenic mice overexpressing Akt [110] and Ski [111], also show decreased fat mass. However, we have recently identified expression of the myostatin receptor in bone marrow-derived mesenchymal stem cells (BMSCs), and found that BMSCs from myostatin-deficient mice demonstrate increased osteogenic differentiation and decreased adipogenic differentiation [112]. There are not many studies examining the effect of myostatin on PPAR-*γ* expression. A study by Artaza et al. [107] demonstrated that myostatin increased expression of C/EBP alpha and adipogenesis in mesenchymal stem cells, suggesting a myostatin effect on PPAR-*γ*, although they did not actually examine PPAR-*γ* expression. These data are consistent with previous reports showing increased bone mineral density in the bones of myostatin-deficient animals [113–115]. Furthermore, these data from bone marrow cells provide further evidence that myostatin is an adipogenic factor, as well as one that suppresses myogenesis and perhaps osteogenesis. In contrast, a study by Hirai et al. [116] found that myostatin inhibited PPAR-*γ* and C/EBP alpha expression in bovine preadipocytes. Thus, myostatin effects on PPAR-*γ* may be cell-type-dependent.

## 8. GLUCOCORTICOID-INDUCED LEUCINE ZIPPER

GILZ, which is also induced by estrogen and sonic hedgehog (Shh), is a new member of the leucine zipper protein [117, 118] and belongs to the TGF-*β*-stimulated clone-22 (TSC-22) family of transcription factors [119, 120]. Members of this family of proteins contain three distinct domains: an N-terminus TSC box, a middle leucine zipper domain, and a C-terminus polyproline-rich domain. GILZ was originally identified from dexamethasone-treated murine thymocytes [118]. Recent studies have shown that GILZ is also induced in many tissues (including lung, liver, brain, and kidney) and by the other glucocorticoids that are prescribed frequently in clinic such as methylprednisolone, fluticasone, and hydrocortisone, as well as anti-inflammatory cytokine interleukin-10 (IL-10) in human and murine macrophages [121–123]. Studies carried out in vitro have shown that overexpression of GILZ protected T cells from apoptosis induced by anti-CD3 antibody, but not other apoptosis-inducing agents such as dexamethasone, various doses of ultraviolet irradiation, starvation, or triggering induced by cross-linked anti-Fas monoclonal antibody [118]. However, T-cell-specific transgenic overexpression of GILZ resulted in thymocyte apoptosis ex vivo possibly through downregulation of Bcl-xL [124]. GILZ also inhibits interleukin-2 (IL2)/IL-2 receptor expression (63). This antiapoptotic function is mediated through direct protein-protein interactions between GILZ and NF-kB, and between GILZ and AP-1 (63–67). The direct interactions of GILZ with NF-kB, and GILZ with AP-1, block DNA binding and, therefore, the transcriptional activities of NF-kB and AP-1.

Studies by Shi et al. [125] found that GILZ is rapidly induced by dexamethasone in MSCs and a variety of cell lines, including osteoblasts (2T3), preadipocytes (3T3-L1), and a mesenchymal cell line (C3H10T1/2). It is interesting to note that the induction of GILZ in MSCs and C3H10T1/2 cells seems transient. GILZ can bind specifically toa 40-bp DNA fragment containing a unique tandemly repeated C/EBP-binding element present in the promoter of the PPAR-*γ*2 gene. Because glucocorticoids induce adipocyte differentiation, and GILZ is induced by glucocorticoids and binds to adipogenic PPAR-*γ*2 promoter, it was hypothesized that constitutive expression of GILZ would activate PPAR-*γ*2 expression and enhance adipogenesis. Contrary to expectations, overexpression of GILZ inhibited PPAR-*γ*2 transcription and blocked adipocyte differentiation of C3H10T1/2 mesenchymal cells and 3T3-L1 preadipocytes. These results demonstrated that GILZ functions as a transcriptional repressor of PPAR-*γ*2. Studies by Zhang et al. (unpublished data) show that overexpression of GILZ in mouse bone marrow MSCs can enhance MSC osteoblast differentiation. These data suggest that, by modulating PPAR-*γ* expression, GILZ may serve as an important regulator of the MSC lineage commitment between osteoblast and adipocyte. This role of GILZ may have potential clinic importance since as humans age, the number of adipocytes increase and the number of osteoblasts decrease resulting in weakened bone and age-related osteoporosis and fragility fractures. All these may have direct connection to the increased PPAR-*γ* expression and activity in aging bone marrow as it is known that aging activates marrow adipogenesis and fat secretes large amounts of cytokines that will, in turn, inhibit osteogenesis as mentioned earlier.

As previously mentioned, the transcriptional activity of PPAR-*γ* is regulated by phosphorylation (by kinases such as the mitogen-activated protein kinase (MAPK), which activate Jun N-terminal kinase, or JNK, and extracellular signal-regulated kinase 2, or ERK 2), and GILZ can directly interact with Raf1, one of the MAPK members, resulting in the inhibition of Raf-1 phosphorylation and, subsequently, the suppression of both MEK/ERK-1/2 phosphorylation and AP-1-dependent transcription [119].

It has been a long standing paradox that glucocorticoids, while required for osteoblast differentiation of primary bone marrow stromal cells in vitro [126–128], induce bone loss in vivo. Since GILZ is induced by glucocorticoids and enhances MSC osteogenesis, we speculate that GILZ is the actual mediator of glucocorticoid action in this process. The possible pathways in which GILZ may convey therapeutic effects of glucocorticoids have been reviewed by Clark and Lasa [129].

Under normal conditions, GC levels fluctuate in response to environmental stressors (flight/fight, abrupt temperature changes, etc.). When the GC level is increased, GILZ is induced and prevents adipogenic differentiation. Under pathophysiological or pharmacological conditions, however, GC is elevated for a prolonged period of time and the negative feedback network is overwhelmed, resulting in harmful GC side effects, such as bone loss.

## 9. GIP, LEPTIN, MYOSTATIN, AND GILZ AS THERAPEUTIC TARGETS

Adequate nutrition and a positive energy balance are clearly important for bone growth. A reduction in caloric intake will retard growth plate expansion [130]. In addition, if the reduced caloric intake is accompanied by a reduced calcium intake, a shift in the balance between bone formation and resorption occurs, such that bone mass decreases over time [131]. In contrast, an increase in intake and gain in weight are associated with an increase in bone mass. Upon nutrient delivery to the intestine, there is a rapid rise in a number of enteric hormones that serve to inform the target tissues that nutrients are available for anabolic activity. One of these enteric hormones, GLP-2, has already been used to prevent bone loss in postmenopausal patients [62], although no data are available on marrow adiposity. Our data would suggest that GIP is another enteric hormone that can increase bone formation by promoting MSC differentiation into osteoblasts.

One of the challenges in using recombinant leptin therapy to either reduce body weight, suppress appetite, or stimulate bone formation is that most individuals are relatively resistant to exogenous leptin treatment due to relatively high levels of endogenous leptin [89, 96]. However, leptin may have significant effects on bone formation and appetite in conditions where leptin sensitivity is increased with energy deprivation. For example, leptin treatment has been observed to increase serum IGF-1 and serum osteocalcin in women with exercise-induced hypothalamic amenorrhea [132]. Anorexia nervosa is associated with markedly reduced leptin levels and osteoporosis [133–135] even if less severe, voluntary weight loss is associated with increased rates of bone loss in adults [136, 137]. Leptin treatment may have potential to reverse bone loss with weight loss, as well as maintenance of reduced weight following weight loss [138].

Treatment of normal rodents and dystrophin-deficient mdx mice with factors that block myostatin signaling, such as a soluble myostatin receptor, a propeptide, or follistatin, showed significant increases in muscle mass and improved muscle regeneration [139, 140]. The myostatin antibody MYO 029 is currently in Phase II clinical trials for treatment of Duchenne muscular dystrophy. To date, myostatin inhibitors have only been tested for their ability to improve muscle regeneration in cases of muscular dystrophy and acute injury, and their potential for inhibiting body fat gain and stimulating bone formation remains relatively unexplored. We expect that myostatin inhibitors have significant potential as novel therapies for decreasing adiposity and also improving bone formation and bone strength. Moreover, as noted earlier in this paper, glucorticoids play a major role in stimulating bone marrow adipogenesis, and the myostatin promoter is known to have a glucocorticoid response element [141]. Myostatin deficiency inhibits muscle atrophy with glucocorticoid treatment [142], and myostatin inhibitors may be useful for attenuating muscle atrophy and bone loss with prolonged use of glucocorticoids.

Bone cells are derived from marrow MSCs, and the best way to increase the number of bone-forming cells is to modulate differentiation pathways so that more MSCs are directed to the osteoblastogenesis pathway. The PPAR-*γ* pathway not only regulates adipocyte differentiation, but also inhibits osteoblast differentiation from mesenchymal progenitors [31], suggesting the possibility of interrupting the PPAR-*γ* pathway as a novel treatment of osteoporosis. GILZ, induced transiently by GC, is a sequence-specific transcriptional repressor of PPAR-*γ* [143]. No transcriptional repressors that can bind specifically to the promoter of PPAR-*γ* have been reported so far. Thus, GILZ may be a novel therapeutic target for drug development for a variety of conditions characterized by an altered adipocyte/osteoblast balance.

In summary, current therapeutic targets for prevention and treatment of osteoporosis involve anabolic agents stimulating osteoblastic activity or antiresorptive agents targeting the osteoclasts. Our data would suggest that modulating the MSC differentiation pathway, particularly via inhibition of the PPAR-*γ*2 receptor, thus favoring osteoblastic instead of adipocytic differentiation, might be an attractive therapeutic target for prevention and treatment of osteoporosis.
